# Radiological findings of unilateral tuberculous lung destruction

**DOI:** 10.1007/s13244-017-0547-4

**Published:** 2017-02-14

**Authors:** Diego Varona Porres, Oscar Persiva, Esther Pallisa, Jordi Andreu

**Affiliations:** Hospital Vall d´Hebrón. Radiology department, Passeig Vall d´Hebrón 119, 08035 Barcelona, Spain

**Keywords:** Tuberculosis, pulmonary, Infections, respiratory, CT scanner, X-ray, Radiography, Thoracic

## Abstract

**Objectives:**

The aim of this report is to identify the radiological findings of unilateral tuberculous lung destruction (UTLD).

**Materials and methods:**

Thirteen patients with (UTLD) were reviewed from 1999 to 2014. Only patients with radiological evidence of absence of pulmonary parenchyma preserved were included. Clinical and demographic data were obtained and radiological studies (chest radiograph and CT) were retrospectively reviewed.

**Results:**

The left lung was more commonly involved (85%). The following radiological findings were found in all cases: a decrease in the diameter of the pulmonary vessels of the affected lung, herniation of the contralateral lung and hypertrophy of the ribs and/or thickening of extrapleural fat. Two radiological patterns were identified: UTLD with cystic bronchiectasis (85%) and UTLD without residual cystic bronchiectasis (15%). Forty-six per cent of cases had respiratory infection symptoms with presence of air-fluid levels in the affected lung as the most common finding in these patients.

**Conclusions:**

Total unilateral post-tuberculous lung destruction is an irreversible complication with the following main radiological features: predominantly left-sided location, decreases in the diameter of the ipsilateral pulmonary vessels, herniation of the contralateral lung and hypertrophy of the ribs and/or thickening of extrapleural fat.

**Teaching Points:**

• *Unilateral tuberculous lung destruction is an irreversible complication of tuberculosis.*

• *Left-side predominance and herniation of the contralateral lung are characteristic.*

• *Decreased diameter of the ipsilateral pulmonary vessels occurred in all patients.*

• *The pattern with residual cystic bronchiectasis is the most frequent.*

• *Superimposed non-tuberculous infections may affect the destroyed lung.*

## Introduction

Tuberculosis (TB) remains one of the world’s deadliest communicable diseases. In 2014, an estimated 9.6 million people developed TB and 1.5 million died from the disease [1]. The appearance of strains resistant to pharmacological treatment and immigrant influx from countries with a high incidence of this disease may increase the possibility of TB-related complications and sequelae in developed areas.

Pulmonary TB can be a source of severe complications, such as unilateral lung destruction occurring in the final phase of progressive disease or disease reactivation. This complication has been previously reported in a recent review of 5,926 cases of TB in 74 patients (1.3%) [2]. Lung destruction, particularly in association with bronchiectasis, is easily colonised by bacteria and fungi [3] and this may be one reason why pulmonary TB is associated with considerable morbidity and mortality [4]. The related literature contains few references to this complication [5–7], also known as the left bronchus syndrome. Pulmonary destruction by TB, particularly total destruction of an entire lung, is currently uncommon, and the imaging features may be confused with those of other entities. Hence, it is important to be familiar with the most significant radiological findings in this condition.

In this report, we present a review of 13 patients with UTLD secondary to TB attended in our centre, with the aim of describing the radiological findings in radiograph and chest CT of this late and uncommon complication of TB.

TUTLD is a late complication that requires years to develop; so it is difficult to estimate the influence of other superimposed nontuberculosus infections and to assemble a large sample or perform a prospective study with significant data. However, for this review article we were able to collect these cases to discuss the typical findings of UTLD against the background of findings in patients of our own department. This instructive patient sample was not obtained from a database containing all tuberculosis cases occurring in the study period; therefore the incidence of this complication in our setting will not be discussed in detail.

## Epidemiology

Among the 13 patients comprising the series, there was a male predominance (9 males and 4 females) and the age range was 27 to 83 years (mean 55 years). All the patients were diagnosed with chest tuberculosis during childhood or youth. Two patients were from countries with a higher incidence of TB than Spain [8] (Table 1).

**Table 1:** Demographic and clinical data of the study patients

	Sex/age	Country of origin	Medical history	TB treatment	Symptoms
1	Female/43 years	Guinea	TB at 24 years	Complete treatment with 4 drugs for 24 months	Fever
2	Male/27 years	Ecuador	TB at 20 years Mother died of TB at 20 years	Not recorded in medical history	Cough with mucus expectoration and left pleuritic pain
3	Male/83 years	Spain	TB in infancy	Not recorded in medical history	Weakness and paresthesia of the hands
4	Female/38 years	Spain	TB at 28 years	Complete pharmacological treatment for 6 months	Sudden dyspnoea due to right tension pneumothorax
5	Female/69 years	Spain	TB at 12 years, reactivation at 55 years Kidney TB	Collapse therapy and intramuscular streptomycin during adolescence. Reactivation 14 years ago treated for 6 months with drugs not specified in medical history	Haemoptysis
6	Female/68 years	Spain	TB at 29 years	Complete pharmacological treatment during patient’s second pregnancy	No symptoms
7	Male/73 years	Spain	TB in youth COPD	Collapse therapy	Fever and purulent expectoration
8	Male/72 years	Spain	TB at 18 years, reactivation at 55 years Long-term COPD	Pharmacological treatment for 12 months at 18 years. Reactivation treated with 3 drugs for 18 months	Dyspnoea, cough and purulent expectoration
9	Male/81 years	Spain	TB at 25 years Long-term COPD	Not recorded in medical history	Dyspnoea, cough, purulent expectoration, chest pain and fever
10	Male/73 years	Spain	TB at 16 years Chronic empyema	Therapeutic pneumothorax	Evaluation for noninvasive mechanical ventilation
11	Male/58 years	Spain	TB at 30 years HIV	Complete pharmacological treatment with 3 drugs for 9 months	Dyspnoea
12	Male/66 years	Spain	TB in infancy COPD	Not recorded in medical history	Dyspnoea and expectoration
13	Female/76 years	Spain	TB at 28 years Sleep apnoea syndrome	Not recorded in medical history	Non-productive cough

TB, tuberculosis; COPD, chronic obstructive pulmonary disease; HIV, human immunodeficiency virus

Regarding symptomatology in these patients, most of them presented with dyspnoea or a respiratory infection event. One patient showed self-limited haemoptysis probably related to the existence of bronchiectasis.

With regard to the anti-TB treatment received, it was not collected in the clinical records of five patients and available for the nine patients remaining. Three patients were treated with collapse therapy, which could have promoted the lung destruction. Tuberculous reactivation was treated with medical treatment in one patient. The remaining five patients (5/13, 38.5%) were treated with anti-TB drugs with tuberculous reactivation in one case treated again with drugs.

Four patients were diagnosed with chronic obstructive pulmonary disease (COPD) (4/13, 31%). The relationship between TB and COPD is well recognised and can increase with age [9]. The mean age of COPD patients in our study was quite high (73.5 years, range 66–81), in keeping with these reported data. A proportional relationship between lung function impairment and the degree of abnormalities in chest X-rays has been reported in a previous study [10]; however, we were unaware of the pulmonary function status of our patients.

## Radiological findings

### Chest radiograph findings

PA chest X-ray showed diffuse opacity of a whole hemithorax with severe ipsilateral mediastinal deviation, mimicking an almost complete lung collapse, in one case. The remaining 12 patients presented with a small hemithorax with aireated lung secondary to contralateral lung herniation and/or cystic bronchiectasis or cavitated lesions. Lateral projection showed retrosternal hyperclarity in all patients in relation to the existence of lung herniation contralateral to the UTLD.

### CT findings

**Fig. 1:**
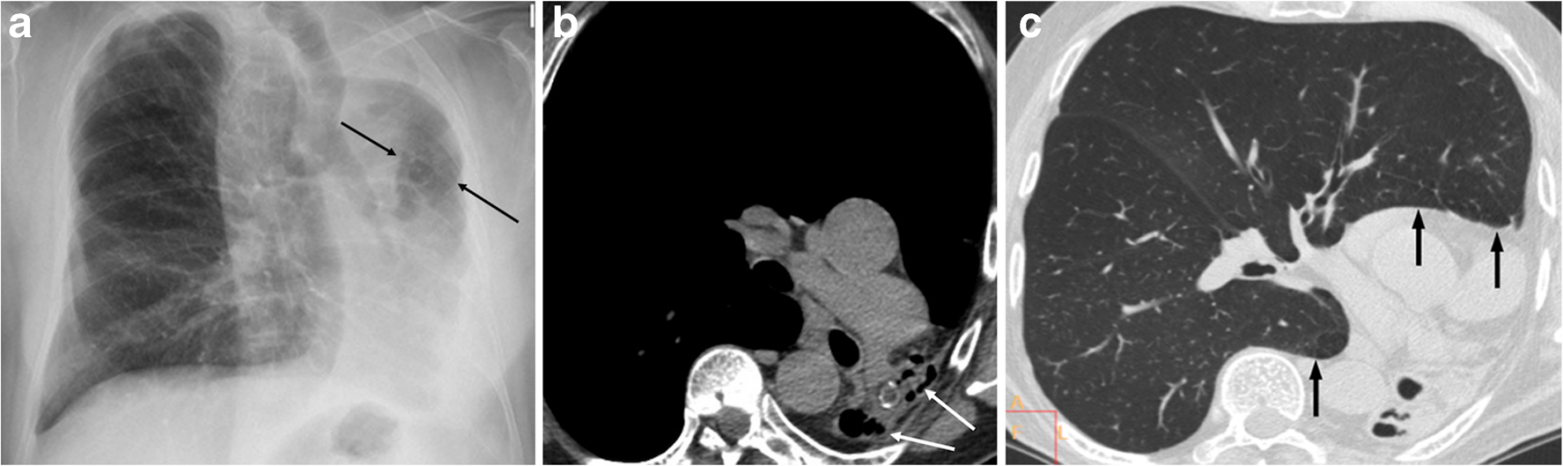
A 58-year-old male with a history of pulmonary TB 20 years previously presented with dyspnoea on exertion. (**a**) The chest radiograph shows marked loss of left lung volume, with an isolated hyperlucent lesion (arrows) that reflects herniation of the contralateral lung toward the left hemithorax. (**b**) Unenhanced multislice CT depicts total destruction of the left lung with residual cystic bronchiectasis (arrows) and herniation of the right lung toward the left hemithorax. (**c**) Unenhanced multislice CT clearly demonstrates herniation of the right lung toward the left hemithorax, that is, anterior herniation of the upper lobe and posterior herniation of the lower lobe (arrows)

**Fig. 2:**
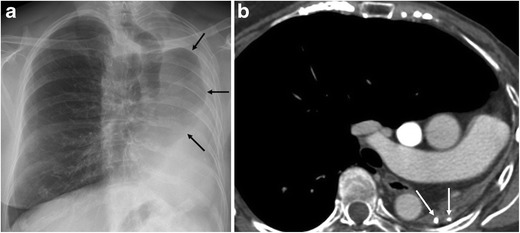
A 68-year-old female with a background of pulmonary TB at age 29 was seen at our hospital for a breast cancer follow-up study. (**a**) Chest radiography shows marked loss of left lung volume and herniation of the contralateral lung (arrows). (**b**) Contrast-enhanced multislice CT demonstrates total left lung destruction with no residual cystic bronchiectasis. Calcifications are seen in the remnant lung (arrows), and the contralateral lung is herniated

**Fig. 3:**
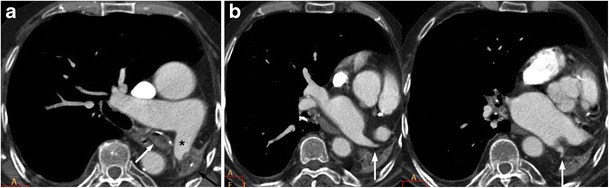
An 83-year-old male who had pulmonary TB in infancy came to our hospital for weakness and paresthesia of both hands. (**a**) On contrast-enhanced multislice CT, total left lung destruction with calcifications (black arrow) in the remnant lung, occupation of the left main bronchus (white arrow) and decreased diameter of the left main pulmonary artery (asterisk) are visualised. (**b**) The diameter of the ipsilateral superior and inferior pulmonary veins is also decreased (arrows)

On retrospective review of the CT, the patients were classifiable in two main patterns: unilateral lung destruction with residual cystic bronchiectasis (11/13, 85%) (Fig. 1) and unilateral lung destruction without residual cystic bronchiectasis (2/13, 15%) (Fig. 2). There was a clear left-sided predominance (11/13, 85%). All patients with UTLD presented three common findings: a decrease in the diameter of the pulmonary artery and pulmonary veins of the affected lung (Fig. 3), anterior and posterior (retrocardiac) herniation of the contralateral lung (Fig. 1c) and hypertrophy of the ribs and/or an increased extrapleural fat. The contralateral lung herniation consisted of anterior herniation of the upper lobe or middle lobe (12/13, 92%) or posterior herniation of the lower lobe (11/13, 85%), with both lobes being affected simultaneously in most cases (10/13, 77%).

### Lung destruction and bronchial tree anomalies

Pulmonary tuberculosis infection leads to lung destruction through a process of cavitation, spread of disease to other areas and secondary development of fibrosis [2].

Lung destruction due to primary progressive TB is generally unilateral, with upper lobe predominance [2]. The presence of peribronchial or hilar lymph adenopathies, a common finding in primary infection that is occasionally accompanied by endobronchial injury, can give rise to bronchial obstruction. The lymph adenopathies may disappear, while the bronchial stenosis persists. Bronchial obstruction due to caseum, pus or an excess of mucus can lead to a secondary pyogenous infection or lobar collapse, with resulting atelectasis due to fibrosis of a lobe (partial) or the entire lung.

Lung destruction due to TB reactivation presents differences relative to the primary disease. It consists of pulmonary dissemination and secondary fibrosis, and the hemithorax contralateral to the infection is often affected [2, 11].

Although the posterior and apical segments of the upper lobes are the most common sites of pulmonary TB, with a similar incidence on both sides, lung destruction due to primary or postprimary disease most commonly occurs on the left side, in the same way as the fibrotic phase of endobronchial TB [3, 12]. This finding was observed in 11 patients in our review (85%), in agreement with other retrospective studies in which left-side predominance ranged from 63% to 80% [4–7, 13]. The explanation resides in the anatomy of the left pulmonary bronchus, which crosses a narrow anatomical space, the aorto-pulmonary window, and is longer and smaller in diameter than the main right bronchus [14]. These factors all favour bronchial collapse due to adjacent lymph adenopathy. In addition, the more horizontal course of the main left bronchus can have an effect on drainage of secretions, favouring obstruction. The CT results in our series support this theory, with left bronchial stenosis as the most common finding.

### Decreased diameter of pulmonary arteries and veins

One radiological finding of note was the clear decrease in the diameter of the ipsilateral pulmonary artery observed in all cases of total lung destruction. This finding is consistent with a reported haemodynamic factor that contributes to bacterial dissemination to the rest of the lung [14]. It consists of shunts between the pulmonary and bronchial arteries, occurring in response to the reduction in pulmonary artery flow associated with bronchial obstruction, an accumulation of secretions, or parenchymal infection. The shunts produce retrograde broncho-pulmonary flow that enhances bacterial dissemination and lung destruction when it is associated with lymphatic stasis and increased oxygen tension in the affected lung [14].

Ipsilateral pulmonary veins also showed a decreased calibre in every case; therefore UTLD should be included in the differential diagnosis of decreased size of pulmonary veins [15].

### Hypertrophy of ribs and thickening of extrapleural fat

**Fig. 4:**
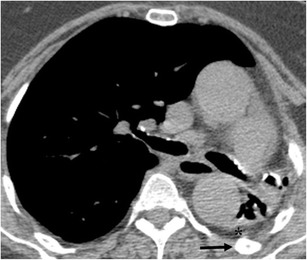
A 76-year-old female with TB history in infancy presenting with dry cough for 1 month. Non-enhanced CT shows total unilateral left lung destruction with bronchiectasis. Upper ipsilateral ribs are hypertrophic (black arrow) and extrapleural fat proliferation is also evident (asterisk)

Hypertrophy of the ribs and/or increased extrapleural fat was present in all patients and is likely a reflection of the lengthy evolution and chronic disease (Fig. 4). Patients with chronic inflammatory conditions of the lung or pleura, especially tuberculosis, may have hypertrophy of the ribs on the affected side. This finding is probably due to chronic reactive periostitis associated with localised hyperaemia [16].

Extrapleural fat thickening is a previously described finding in patients with empyema of the affected side and may increase to up to two to three times more than the contralateral side [17]. In case of thoracic tuberculosis, it has been described in chronic empyema and fibrothorax and is considered a finding suggesting chronicity [11].

### Superinfections

**Fig. 5:**
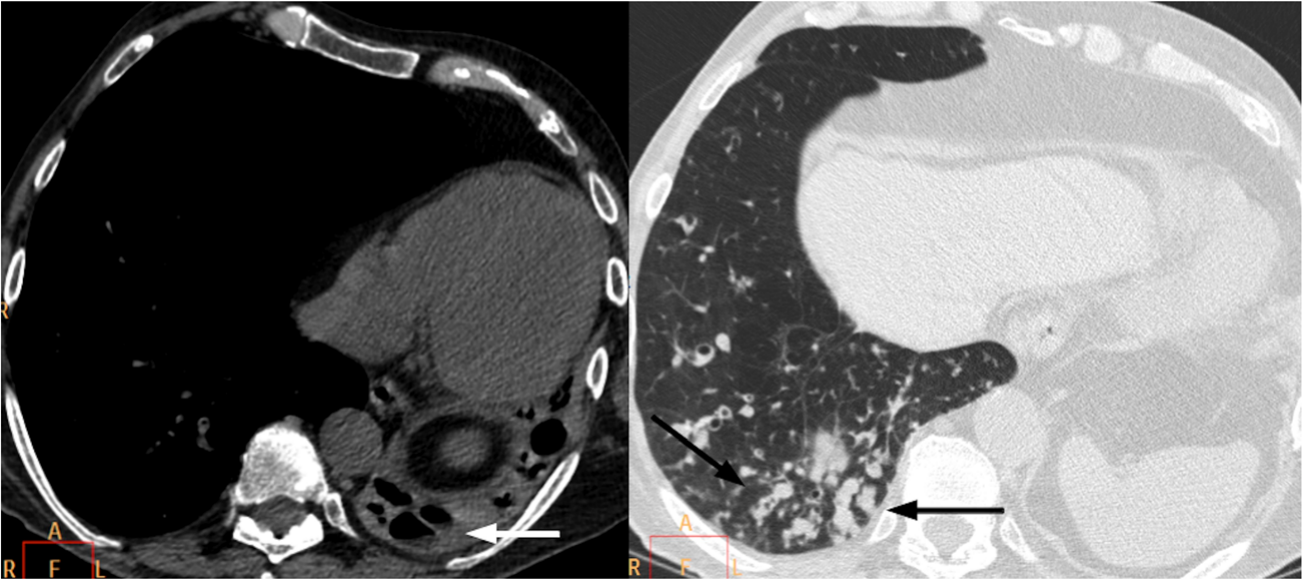
A 73-year-old male with a history of pulmonary TB as a youth and chronic obstructive pulmonary disease was seen for fever and purulent expectoration. Unenhanced multislice CT depicted complete left pulmonary destruction with residual cystic bronchiectasis associated with air-fluid levels (white arrow in the right image) and airway occupation with mucus impaction in the lower right lobe (black arrows in the left image). Sputum culture was positive for *Pseudomona aeruginosa* and *Streptococcus pneumoniae*. Antibiotic treatment was established without success, and the patient died 15 days after hospitalisation

Six patients (6/13, 46.1%) had symptoms consistent with respiratory infection (fever and expectoration), and four showed the following radiographic findings: pulmonary air-fluid levels (2/6, 33.3%), pulmonary air-fluid levels and mucus impaction (1/6, 16.6%) (Fig. 5) and centrilobular pulmonary nodules (1/6, 16.6%). In four patients, microorganisms were isolated from sputum samples or bronchoalveolar lavage: *Pseudomona aeruginosa* (3/4, 75%), *Streptococcus pneumoniae* (1/4, 25%) and *Haemophilus influenzae* (1/4, 25%).

Tuberculosis infection tends to be inactive in cases of lung destruction, making the diagnosis more difficult. The microbiological study of secretions often shows other microorganisms [2], which may be of bacterial origin, such as those described in our review and indicating processes of superinfection. The isolated microorganisms in our series (*Pseudomonas aeruginosa, Streptococcus pneumoniae*, and *Haemophilus influenza*) are consistent with the findings from a prospective study in hospitalised patients with acute COPD exacerbations [18] and similar to previous literature [19]. All COPD patients in our study had symptoms suggestive of respiratory infection and also had positive cultures or positive radiology; hence, the presence of respiratory superinfection in our patient sample with UTLD might be falsely increased because of the association with COPD. The incidence of respiratory infections may be falsely increased because of the presence of COPD, as it has been reported that respiratory infections cause the majority of COPD exacerbations [20].

In patients treated in our hospital, we observed two radiological patterns: unilateral destruction with cystic bronchiectasis and unilateral destruction without cystic bronchiectasis. All patients with superinfections signs had the total destruction with cystic bronchiectasis pattern. Therefore, the pattern of lung destruction with cystic bronchiectasis is associated with the highest risk of superinfection.

### Other findings

**Fig. 6:**
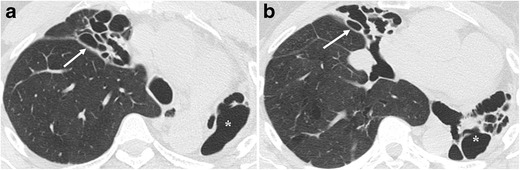
A 43-year-old female with TB history in her youth and recurrent infections presenting with fever. Sputum culture was postive for *Pseudomona aeruginosa*. (**a**) Non-enhanced CT shows total unilateral left lung destruction with bronchiectasias (asterisk) and lung atelectasis with saccular bronchiectasis in the right upper lobe (white arrow). (**b**) Right middle lobe atelectasis is also seen along with bronchiectasis (white arrow)

**Fig. 7:**
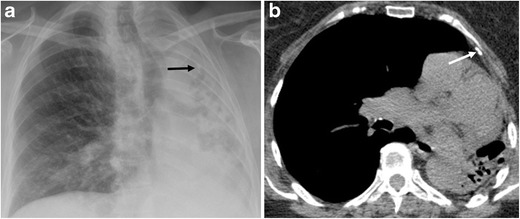
Same patient as in Fig. 4. (**a**) Chest radiograph shows total unilateral left lung destruction and calcification projected on the upper half of left hemithorax. (b) Non-enhanced CT confirms the presence of left upper pleural calcification in correspondence with the calcification seen on chest radiograph

The contralateral lung in post-tuberculous lung destruction showed residual lesions, such as bronchiectasis (Fig. 6), bronchial thickening, calcified granulomas and atelectasis in ten patients (10/13, 78%). Pulmonary, mediastinal, pleural, pericardial and abdominal calcifications were seen in ten patients (10/13, 78%) and were visible on plain films in four of them (Fig. 7).

## Differential diagnosis

The diagnosis of this entity is straightforward if the previous history of TB is clear and well documented on clinical records. When radiological findings consistent with lung destruction are seen, we should consider any granulomatous disease. Similar features can be observed in some types of mycosis, although the fibrosis and mediastinal displacement are never as severe as in TB [2]. The differential diagnosis should also include total lung collapse, chronic pleural disease, previous lung surgery and pulmonary agenesis/hypoplasia. Pulmonary agenesis/hypoplasia can affect both lungs and may be indistinguishable from UTLD. In most cases, however, the diameter of the pulmonary artery is normal [21] and this contrasts with the smaller diameter of the pulmonary artery seen in all patients with UTLD. UTLD should be included in the differential diagnosis of causes of decreased calibre pulmonary arteries and veins. Residual lesions in the contralateral lung and calcifications were present in a high percentage of patients in our series and can also help to support the diagnosis of post-tuberculous lung destruction along with the clinical history of previous lung tuberculosis.

## Management

Several papers in the medical literature describe surgical treatment of the UTLD by means of pneumonectomy in certain cases, though this treatment is considered high risk [13, 22–24]. Cases of postpneumonectomy-like syndrome have also been described in patients with UTLD; accordingly this condition should be included in the differential diagnosis of dyspnoea in this group of patients [25, 26].

In our daily practice all patients were incidentally diagnosed as a consequence of signs and symptoms of respiratory infection or dyspnoea. Due to the appearance of infections in these patients an imaging examination may be necessary for the diagnosis.

## Conclusion

In conclusion, UTLD is an irreversible complication of pulmonary TB that presents with characteristic radiological findings: unilateral volume loss predominantly in the left lung, decreased diameter of the ipsilateral main pulmonary artery and pulmonary veins, and herniation of the contralateral pulmonary parenchyma. The pattern with residual cystic bronchiectasis is the most frequent. Usually the tuberculous infection is inactive, although associated non-tuberculosis infection may develop and affect the destroyed lung. Therefore, a correct diagnosis and radiological follow-up are very important in these patients.
